# Does Aleutian Disease Occur among Domestic Ferrets in Poland? Results of Preliminary Studies Conducted in Two Regions of Poland

**DOI:** 10.3390/ani12192673

**Published:** 2022-10-05

**Authors:** Alicja Blank, Paweł Foksiński, Joanna Małaczewska, Mirosława Blank, Anna Rzepka, Andrzej Krzysztof Siwicki, Roman Wójcik, Edyta Kaczorek-Łukowska

**Affiliations:** 1 Department of Microbiology and Clinical Immunology, Faculty of Veterinary Medicine, University of Warmia and Mazury in Olsztyn, Oczapowskiego 13, 10-719 Olsztyn, Poland; 2 Association of Friends of Ferrets, Mickiewicza 18a/4, 01-517 Warsaw, Poland; 3 PULSVET Specialist Veterinary Clinic, Alternatywy 7/U8, 02-775 Warsaw, Poland

**Keywords:** ferrets, Aleutian disease, Parvoviridae, Carnivore *amdoparvovirus*

## Abstract

**Simple Summary:**

Aleutian disease (AD) is a well-known viral disease among mink breeders; however, it has the potential to transmit to other species, including ferrets. There is little research on the presence of AD among ferrets, especially those kept as house-pets. Our study determined the percentage of actively infected animals and those that had specific antibodies to the AD virus. Among the animals studied, we also had those that had contact with minks with confirmed Aleutian disease. The results allow us to conclude that although the virus is present in the Polish ferret population, it rarely causes a fatal disease, rather causing non-specific symptoms. We believe that future studies involving a larger number of animals, as well as more accurate testing methods, will allow us to determine more accurately how serious Aleutian disease is among ferrets.

**Abstract:**

Although ferrets are becoming increasingly popular as companion animals, their population in households is still far lower compared to cats or dogs. This results in a much smaller number of ferret specialists, and thus poorer diagnosis of various diseases, including the Aleutian disease. Aleutian disease is a slowly progressing viral disease which can cause different symptoms in these animals. The virus can also cause symptoms in different species of animals, but in the case of ferrets, there is relatively less information on about both the prevalence and symptoms of this disease. Therefore, the aim of this study was to determine the presence of antibodies and the virus itself in ferrets from two regions of Poland. Blood samples and rectal swabs were obtained from 61 domestic ferrets from Mazowieckie and Dolnośląskie voivodships. The presence of antibodies was determined using serological methods and real-time PCR analysis was performed to determine presence of viral DNA. Serological analyses demonstrated that 49% (*n* = 30) of the ferrets had antibodies against Aleutian disease virus (ADV). No relationship was observed between the prevalence of antibodies and age, sex, habitual residence or origin of ferrets. The real-time PCR did not confirm DNA of the ADV in any of the blood and rectal swab samples. Obtained results suggest that ADV circulates in the analyzed population of ferrets, therefore further studies in this direction should be carried out.

## 1. Introduction

Despite the growing popularity of ferrets as pets, their population is still relatively small compared to other more frequently chosen species of companion animals, such as dogs and cats. Their lesser popularity results in a much smaller number of ferret specialists, and thus poorer diagnosis of various diseases, including the Aleutian disease. The Aleutian disease virus (ADVwas reclassified in 2014 as carnivore amdoparvovirus belonging to the Parvoviridae family, genus *Amdoparvovirus* [1] and is a naked icosahedral virus containing single-stranded DNA with three non-structural (NS1, NS2, NS3) and two structural (VP1, VP2) proteins [2]. The strain that causes the Aleutian disease in ferrets, which is Aleutian ferret disease virus (AFDV), is a mutant of the mink strain, Aleutian mink disease virus (AMDV) and is 90% antigenically homologous [3,4]. Yet, both strains show species specificity for the ability to induce the fully symptomatic disease [5]. In practice, this means that both strains (AFDV and AMDV) can cause clinical signs [6] in both species of animals. Clinical signs of the disease in ferrets are usually much less pronounced (regardless of the type of virus) than in minks; however, sometimes mink can be fully asymptomatic [7]. Aleutian disease is a slowly progressing viral disease. After infection, the virus circulates in the body in the form of immune complexes [8,9], which deposit in the blood vessels of organs, leading to their damage, which causes the late onset of symptoms and complicates both correct diagnosis and treatment. It is presumed that phagocytic macrophages of ADV-antibody complexes reactivate the virus, thus allowing it to further replicate in phagocytic cells, which may promote the persistence of the virus in the body, and an important factor in its virulence [10]. In postmortem studies, viral DNA is most often found in ferret’s spleen, lung, liver, and kidney specimens, and in pericardial fat [7], while the presence of the virus in vivo is confirmed by testing blood, saliva, and rectal swabs [11]. Regardless of the type of sample taken, viral DNA is detected using the PCR method. The ADV infection most often occurs through the contact with infected secretions and excreta, with contaminated every-day objects at home or at exhibitions and animal markets [4]. Minks can also become infected vertically. It is assumed that in ferrets, the AFDV also has the ability to pervade the placental barrier [4] and accumulate in the ovaries and mammary glands. The most common sign of ADV infection in ferrets is hypergammaglobulinemia, at the level of >20% of total globulins. Other clinical signs include worse mood, weight loss, the appearance of tarry feces, and chronic wasting [12]. However, despite the presence of the virus, most ferrets may not develop clinical signs at all. The virus may exist in the body in a latent form and be activated as a result of stress or immunosuppression [4].

Although Aleutian disease in ferrets was described as early as in the 1960s [13], knowledge about it is still insufficient due to its difficulty to diagnosis. Screening for ADV in ferrets allows both the extension of knowledge and the development of prevention programs. To the best of the authors’ knowledge, no such research has been carried out in Poland so far. Therefore, the aim of this study was to determine the presence of antibodies and the viral DNA in ferrets from two regions of Poland.

## 2. Materials and Methods

Blood samples and rectal swabs were obtained from 61 domestic ferrets (31 males, 30 females) living under the care of both the Ferret Friends Society and private owners from Masovian and Lower Silesian voivodships. The animals were obtained from 28 households and were between 10 months and 8 years old. The number of animals per household ranged from 1 to 8. The test was voluntary, and the samples were taken when veterinarians performed routine, annual morphological and biochemical tests on the animals. The samples were collected from October 2020 till March 2021. On the day of collection, 37 animals were in good health, the remaining 24 showed various disease symptoms described in Table 1. According to a questionnaire completed by owners, 4 of the ferrets analyzed had contact with mink.

Blood (0.5 mL) was collected from the cephalic vein into EDTA test tubes and a test tube with a clotting activator. Swabs were taken from the rectum. The presence of antibodies was determined using the Mink Aleutian Disease Antibody Rapid Test Kit (Ring Biotechnology, Beijing, China), intended for use with minks and ferrets. Viral DNA from EDTA blood and rectum swabs was extracted by using the QIAamp cador Pathogen mini kit (Qiagen, Hilden, Germany) according to the manufacturer’s instructions. An internal control was added to all isolated samples to verify that the genetic material was isolated correctly. Real-time PCR analysis was performed using an Aleutian Disease Virus Advance Kit (PrimerDesign, York House, Chandler’s Ford, UK) according to the manufacturer’s protocol. This kit detects the NS1 gene. Producers of both kits declare sensitivity at the level > 95% and positive control for real-time PCR analysis was delivered by the manufacturer. We used RNase-free water as a negative control in real-time PCR test. In accordance with the manufacturers’ recommendations for the assay, a standard curve was made from the positive sample in the range from 2 × 10^5^ to 2 copy number/µL. Amplification reactions were carried out with a LightCycler^®^ 96 Real-Time PCR thermocycler (Roche, Meylan, France). For the rapid antibody test, positive serum from a ferret provided from a collaborative veterinarian was used as a control sample (archival sample from a ferret not included in the study with the presence of ADV confirmed post-mortem in a commercial lab). A negative control for the strip test was conducted using serum from a healthy mink, which was tested for Aleutian disease by its owner.

## 3. Results

Serological analyses demonstrated that 49.18% (*n* = 30) of the ferrets had antibodies to ADV. Of the 30 individuals that tested positive for antibodies, 43.33% (*n* = 13) showed various symptoms (Table 2), including mainly cough (*n* = 4) and splenomegaly (*n* = 3), which was confirmed by an ultrasound examination. The remaining antibody-positive ferrets were in good health.

Of the four ferrets that came into contact with mink with confirmed Aleutian disease (confirmed by the veterinarian based on existing symptoms and the results of an anti-ADV antibody test), three had antibodies to ADV. No relationship was observed between the prevalence of antibodies and age, sex, habitual residence and origin of ferrets (adoption via Ferrets Friends Society (SPF), pet-shop, registered and unregistered breeding).

The real-time PCR did not confirm DNA of the ADV in any of the blood or rectal swab samples.

It is also worth mentioning the situation of one of the ferrets originating from one household (*n* = 3) analyzed in this study, where one of the ferrets was confirmed to have the antibodies, and the other two were not. Neither of these ferrets were positive in the real-time PCR test, yet the ferret with the antibodies died a few months after the test, showing clinical signs typical for Aleutian disease in ferrets (weight loss, deterioration of the coat quality, enlarged spleen, lethargy, and paralysis). Necropsy revealed histopathological lesions specific to this disease (plasma cells infiltration in liver, kidneys, lymph nodes, pancreas, small intestine and myocardium). On the basis of the obtained necropsy results, the attending veterinarian diagnosed the Aleutian disease. After re-testing the remaining animals in the population, it turned out that one of them developed ADV antibodies.

## 4. Discussion

For many years, it was believed that Aleutian disease affects only ferrets and minks, but there is more and more information that this virus occurs in other animal species, such as: ermine, marten, and lynx [14,15]. Alexanderesen et al. [16] demonstrated that experimental infection with specific antibodies developed was possible in dogs, raccoons, cats, mice, rabbits and foxes, but viral replication occurred only in raccoons and dogs. None of the animals developed symptoms characteristic of the disease, but the species barrier may break over time; hence, the monitoring of the virus is important. Interestingly, ADV infection was also confirmed in humans, in two workers on mink farms [17], with acute clinical symptoms (diarrhea, fever, vomiting, apathy, weakness and high levels of CSF protein), resulting in the production of specific antibodies against ADV. The possibility of viral replication in human tissues is a controversial topic–viral DNA was found in only one patient [17]. Although the described cases of the disease were sporadic, monitoring Aleutian disease in both minks and ferrets seems justified, as the presence of a larger population of these animals in close proximity to humans may lead to a permanent break of the species barrier. On the other hand, in our opinion, of more concern is the fact that this virus can infect many carnivores (including canine-fox and feline-lynx) and may pose a threat to both domestic animals and endangered wild species [15].

When minks are infected with a ferret strain, the characteristic symptoms of the Aleutian disease developed in less than 4% of the tested animals, but specific antibodies were produced in all the tested animals [18]. Our study showed that out of the 4 ferrets that had direct contact with Aleutian-positive mink, three developed specific antibodies against ADV. Compared to the study by Porter et al. [18] who obtained 42% positive results among the animals tested by performing a direct and indirect immunofluorescence test, our results are slightly higher (75%). This could be due to having a smaller group of test animals or the fact that antibody titers dropped 150 days after exposure, according to these studies.

We found antibodies in 44% of individuals who had no contact with ADV-positive mink, which corresponds to the results of studies by other authors [19,20], who noted a relatively high percentage of both free-living minks and other animals (e.g., weasels or striped skunk) with anti-ADV antibodies. The results of the present study confirm that the ADV can be common in the environment. In the case of this virus, the production of specific antibodies after contact with the pathogen does not provide immunity, so these results may be concerning, as they suggest the presence of this virus in the area we analyzed, which is associated with increased morbidity in these animals in the future. Nevertheless, it should be mentioned here that we used rapid tests to determine the presence of antibodies, which can give false-negative results. In addition, false-positive results are possible due to cross-reactivity with other parvoviruses. Domestic ferrets in Poland are vaccinated with a single preparation for infectious diseases such as distemper, parvovirosis and others every 12–18 months. The virus that causes parvovirosis and Aleutian disease belongs to the same family (Parvoviridae), which makes it possible to assume the possibility of developing cross-resistance in animals. In the group of tested individuals, 40 were vaccinated against the above-mentioned diseases, of which 17 (43%) developed specific antibodies. Such a result does not allow us to conclusively confirm that there is such a relationship. Further studies using other methods such as ELISA or counter-current immunoelectrophoresis should be performed [15]. Of all the ferrets tested, 49% developed specific anti-ADV antibodies, yet none had viral DNA, which may indicate the presence of the virus in the latent phase or sampling in the non-viremia state. As suggested by many authors, the most reliable test material for Aleutian disease in mink is the spleen [21,22]. In the case of our study, samples were taken from live animals and therefore only blood samples and rectal swabs were taken; therefore, detection of viral DNA may have been limited. In ferrets, there is little research that has determined where the virus best replicates, especially concerning asymptomatic individuals. Nonetheless, it may be that the virus is latent in the body or has a different replication site than in mink and this may be confirmed by the situation wherein the case described above, where despite a negative result in the real-time PCR test and a positive result in the antibody test, the animal died several months later with symptoms characteristic of Aleutian disease (section performed in a commercial laboratory at the request of the animal owner). This situation suggests that the obtained results may be a false negative and that testing for ADV should be repeated after some time, because viral DNA could be below the detectability level of the diagnostic kit used. Furthermore, we cannot exclude the possibility that some new variant of the ADV virus has appeared among the ferrets we analyzed, which was not detected by the real-time PCR test we used. Nonetheless, further research in this direction should be done using other primer and probe pairs.

Compared to mink, where Aleutian disease has been studied for decades due to its economic aspect, the disease in ferrets is described much less frequently. Over the years there have only been a few studies addressing the problem of spontaneous Aleutian disease in ferrets [23,24,25].

## 5. Conclusions

Based on the obtained results, it can be concluded that Aleutian disease virus is circulating among the population of domestic ferrets in Poland. Although in most of the analyzed animals no characteristic symptoms of this disease were observed, we do not exclude that this phenomenon will not increase in the future. Therefore, further monitoring studies and preventive measures are necessary, especially since there are no effective treatments at this time.

## Figures and Tables

**Table 1:** Health condition of ferrets on the day of examination and results of PCR and antibody tests for Aleutian disease in ferrets (*n* = 61) from two provinces in Poland.

ID	Household Number	Sex	Age (Years)	Antibodies	Real-Time PCR Blood	Real-Time PCR Rectal Swab	Vaccination against Parvovirosis	Health Status during the Examination	History of Disease in the Last Year
1	1	M	3	+	−	−	+	Healthy	None
2	1	M	3	+	−	−	+	Healthy	None
3	2	F	6.5	−	−	−	−	Healthy	None
4	3	F	3.5	−	−	−	+	Healthy	None
5	3	F	3.5	+	−	−	+	Healthy	Enlarged nodes in the abdominal cavity and spleen
6	3	M	2.5	−	−	−	+	Healthy	None
7	4	M	1.5	−	−	−	+	Healthy	Inflammation of the bladder and ear
8	4	M	4.5	−	−	−	+	Healthy	None
9	4	M	3.5	−	−	−	+	Healthy	None
10	5	F	6	−	−	−	+	Healthy	Chronic diarrhea
11	6	F	3.5	−	−	−	+	Healthy	None
12	7	F	5	+	−	−	−	Heart murmurs	None
13	7	F	4	+	−	−	−	Healthy	None
14	8	F	5.5	−	−	−	+	Healthy	None
15	9	F	8	+	−	−	−	Epizootic Catarrhal Enteritis	None
16	9	M	3	+	−	−	−	Healthy	None
17	9	M	5	−	−	−	−	Lymphoma	Epizootic Catarrhal Enteritis
18	9	F	7	+	−	−	−	Epizootic Catarrhal Enteritis	None
19	10	F	6.5	+	−	−	+	Splenomegaly	Kidney failure
20	11	F	4	+	−	−	+	Chronic cough	Chronic cough
21	12	M	6	+	−	−	+	Inflammatory bowel disease	Epizootic Catarrhal Enteritis
22	12	M	6	+	−	−	+	Healthy	Inflammation of the kidneys and gingivitis
23	13	F	7	+	−	−	−	Splenomegaly	Splenomegaly
24	13	F	7	+	−	−	+	Healthy	Cough
25	14	F	4	−	−	−	+	Healthy	None
26	14	F	1.5	+	−	−	−	Healthy	Gingivitis
27	13	M	5	+	−	−	−	Healthy	None
28	15	F	4	+	−	−	−	Healthy	None
29	16	M	5	−	−	−	−	Healthy	None
30	16	M	5	+	−	−	+	Asthma	None
31	13	M	3	−	−	−	+	Healthy	Periodontitis, a tumor on the spleen
32	13	M	5	−	−	−	+	Healthy	Enlarged pancreas
33	13	F	3	−	−	−	+	Healthy	Bronchitis
34	13	M	5	−	−	−	−	Healthy	None
35	13	M	4	−	−	−	−	Healthy	None
36	13	M	5	−	−	−	+	Insulinoma	Tumors on the liver, spleen, pulmonary fibrosis
37	17	F	5	−	−	−	+	Healthy	None
38	17	M	3	+	−	−	+	Insulinoma	Cough, fatty liver
39	17	F	2	−	−	−	+	Healthy	None
40	18	M	2.5	+	−	−	+	Healthy	None
41	18	M	2.5	−	−	−	+	Healthy	Splenomegaly
42	11	M	3	+	−	−	+	Healthy	None
43	11	M	5	−	−	−	+	Healthy	Splenomegaly
44	19	M	4.5	+	−	−	−	Healthy	Bradycardia
45	19	M	2.5	−	−	−	−	Health	Chronic fatigue
46	20	F	4	−	−	−	+	Healthy	None
47	20	M	3	−	−	−	+	Healthy	None
48	21	F	6	+	−	−	+	Inflammation of the liver and biliary tract	Recurrent vomiting
49	22	F	3	+	−	−	+	Cardiomyopathy, insulinoma, nephritis	Inflammation of the upper respiratory tract, insulinoma
50	23	M	5	−	−	−	+	Paralysis	Inflammation of the upper respiratory tract
51	23	M	4	−	−	−	−	Healthy	None
52	24	F	1.5	−	−	−	+	Healthy	None
53	25	F	3.5	−	−	−	+	Healthy	Periodontitis
54	25	F	5.5	+	−	−	+	Healthy	Urinary tract infection
55	26	M	4.5	+	−	−	−	Healthy	None
56	26	F	4	+	−	−	−	Adrenal hyperplasia	None
57	26	M	4	−	−	−	+	Healthy	None
58	26	M	5	+	−	−	+	Healthy	None
59	27	F	2	+	−	−	+	Healthy	None
60	27	F	3	−	−	−	−	Healthy	Splenomegaly
61	28	F	10 months	+	−	−	+	Inflammatory bowel disease	None

**Table 2:** Results of PCR and antibody tests for Aleutian disease in ferrets (*n* = 61) from two provinces in Poland.

Ferrets with Positive Antibodies		qPCR Blood (*n* = 61)	qPCR Swab (*n* = 61)
49.18% (*n* = 30)		0.00%	0.00%
With clinical signs	Without clinical signs		
43.33% (*n* = 13)	56.67% (*n* = 17)		

## Data Availability

The data presented in this study are available in the article.
